# Effect of Exercise Intervention on Flow-Mediated Dilation in Overweight and Obese Adults: Meta-Analysis

**DOI:** 10.1155/2017/7532702

**Published:** 2017-10-01

**Authors:** Younsun Son, Kyungun Kim, Soeun Jeon, Minsoo Kang, Sukho Lee, Yoonjung Park

**Affiliations:** ^1^Department of Health & Human Performance, University of Houston, Houston, TX, USA; ^2^Department of Kinesiology and Health Education, University of Texas, Austin, TX, USA; ^3^Department of Counseling, Health, and Kinesiology, Texas A&M University-San Antonio, San Antonio, TX, USA; ^4^Department of Health and Human Performance, Middle Tennessee State University, Murfreesboro, TN, USA

## Abstract

The objective of this meta-analysis is to summarize the effect of exercise intervention on flow-mediated dilatation (FMD) in overweight and obese adults. We searched four electronic databases (PubMed/Medline, Scopus, and CINAHL) through June 2016 for relevant studies pertaining to the effectiveness of exercise intervention on FMD. Seventeen of the 91 studies identified met the inclusion criteria. Comprehensive Meta-Analysis software (version 3) was used to compute the standardized mean difference effect size (ES) and 95% CI using a random effects model. We calculated 34 ESs. We found that exercise intervention had medium and positive effects on FMD, with an overall ES of 0.522 (95% CI = 0.257, 0.786). Heterogeneity of ESs was observed (*Q*_*b*_ = 239, *p* ≤ 0.001, *I*^2^ = 86.19), and the effect was moderated by comorbidity (*Q*_*b*_ = 6.39, df = 1, *p* = 0.011). A large ES for the combination exercise, low intensity exercise, and comorbidity subgroups (ES = 0.82~1.24) was found. We conclude that while exercise intervention significantly improves FMD in overweight and obese adults, the effect may depend on the different characteristics of exercise intervention and on participants' demographics.

## 1. Introduction

Obesity, a chronic metabolic disorder, is strongly associated with morbidity and mortality as well as a reduced life expectancy [1]. Obesity is defined as an excessive accumulation of adipose tissue. Globally, 1.9 billion adults are overweight or obese, and this figure has more than doubled in the past two decades [2]. Epidemiological studies show that overweight and obese status in adults significantly increases the risks of numerous cardiovascular and circulatory disorders, for example, hypertension, stroke, coronary artery disease, and heart failure [1, 3]. Some of the complex, interrelated pathological states, for example, altered lipid profile and elevated blood pressure, also associated with obesity, subsequently induce insulin resistance, vascular oxidative stress, vascular endothelial dysfunction, and other debilitations [4–6].

The vascular endothelium, which is a single layer of cells lining the interior surface of blood vessels, plays a key role in vasomotor regulation mainly through the nitric oxide- (NO-) dependent signaling pathways [7]. The vascular endothelium is a sensitive structure which is susceptible to damage by certain lipids and inflammatory mediators [8]. Adipose tissue is well known to be associated with inflammatory processes, and it is also implicated in the production of reactive oxygen species (ROS) [9]. Multiple studies provide evidence that vascular endothelial function (EF) is impaired in the setting of obesity [6, 10, 11]. Endothelial dysfunction is considered to be an early precursor and common pathological feature of vascular diseases [12].

Endothelial dysfunction is commonly evaluated by flow-mediated dilatation (FMD) in human studies. The FMD is a noninvasive clinical tool that measures shear stress-mediated vasodilatory response and depends on NO bioavailability [7, 13]. One meta-analysis has reported an association between a 1% decrease in FMD and an 8% increase in the risk of future cardiovascular events [14]. While pharmacological intervention is often used to improve EF or body weight (e.g., topiramate and metformin), regular exercise training is a promising nonpharmacological option in obesity treatment [15].

Although a recent meta-analysis demonstrated the beneficial effect of exercise training on FMD in both obese and nonobese adults [16], the included studies pool data from both obese and nonobese groups, thus raising the question of whether obesity status is a confounding factor in accurate evaluation of FMD. Furthermore, the optimal intensity, modality, and duration of exercise for improving FMD are controversial [17–20]. These and similar studies also suggest there are several other potential factors confounding measurement of FMD in the setting of obesity.

To address the inconclusive findings, we conducted a meta-analysis to quantitatively evaluate the relationship between exercise training and EF in overweight and obese adults. We compared the effects of different characteristics of exercise interventions and participants' demographics on FMD.

## 2. Methods

### 2.1. Literature Search

Source of data was identified by keyword searched from four electronic databases: the PubMed/Medline, Scopus, and CINAHL. The keywords used to identify the relevant studies were “obesity”, “overweight”, “exercise”, “training”, “flow mediated dilatation”, “flow mediated dilation”, and “FMD”. Additional potential sources were identified by hand search using personal databases and a reference list of published studies.

### 2.2. Inclusion and Exclusion Criteria

The studies were included in the review if sufficient information was reported that allowed us to compute the standardized mean difference of FMD. Specific inclusion criteria for eligible studies were the study (1) included the value of relative FMD; (2) included exercise intervention at least 7 days; (3) considered only overweight and/or obese adults; and (4) is written in English language and published in peer-reviewed journals through June 2016. Furthermore, studies were excluded if they were purposefully designed for examining the effects of weight loss medication, antiandrogens, fertility treatments, glucocorticoids, or oral contraceptives.

### 2.3. Coding and Data Extraction

The two authors (YS, SJ) independently coded the identified studies using extraction sheets. The characteristics of the studies were coded for descriptive purposes and moderator analyses. Based on the procedures recommended by Lipsey and Wilson (2005), the outcome and moderator variables were extracted. The effect size (ES) of outcome variable, FMD, was computed using (a) before and after mean difference from intervention groups divided by pooled standard deviation (SD) and (b) mean difference between intervention and control groups divided by pooled SD. Also, moderator variables which may affect overall ES of FMD were coded as follows: body weight change, diet intervention, exercise duration/type/intensity, comorbidity, and baseline Body Mass Index (BMI). Exercise intensity and type were classified as low, moderate, and high intensity using the definition of the American College of Sports Medicine [21].

**Figure 1:**
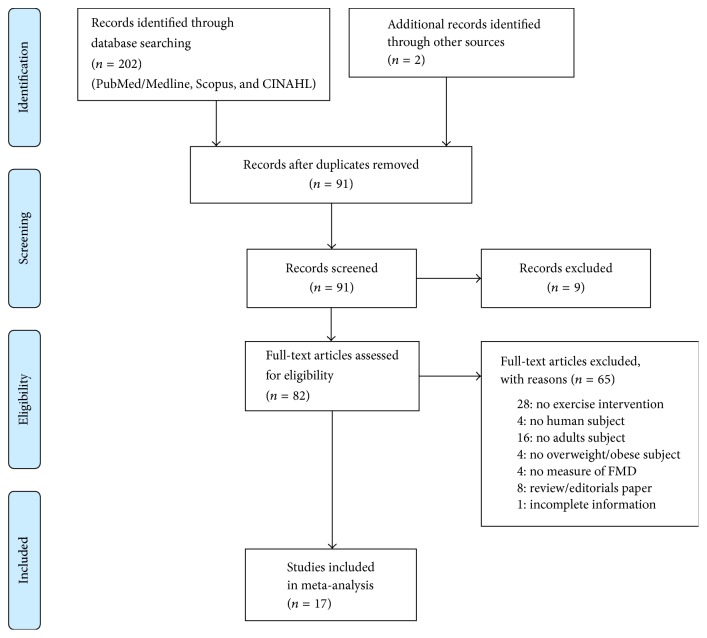
Flowchart for selection of studies.

All coded data were crosschecked with authors for establishing consistency, and discrepancies were resolved by discussion.  Figure 1 illustrates the schematic flow diagram of this study describing the inclusion and exclusion procedures for study selection [22].

### 2.4. Study Quality Rating

The methodological quality of selected studies was assessed using the Physiotherapy Evidence Database (PEDro) scale [40, 41]. This scale consists of 11 items: random allocation, concealed allocation, similarity at baseline, subject blinding, therapist blinding, assessor blinding, >85% follow-up for at least one key outcome, intention-to-treat analysis, between-group statistical comparison for at least one key outcome, and point and variability measures for at least one key outcome. The quality of studies is determined based on the average of overall scores (range = 0–10; each item, except for item 1, contributes one point) where higher scores indicate better methodological quality. The average total PEDro score is 5.0 ± 1.6 (mean ± SD) based on 27,444 records from the PEDro database on January 2017. The scoring ≥ 6/10 was considered “moderate to high” for methodological quality [42].

### 2.5. Data Analysis

All analyses were run in Comprehensive Meta-Analysis version-3 software with a significance level of 0.05. Because we assumed that the variety of research designs with study characteristics might affect the true ES from one study to another, a random effects model was used to estimate the overall ES and 95% confidence intervals (CIs). The measure of ES used for the present study is the standardized mean difference, Cohen's* d*. The ESs were evaluated based on Cohen's guideline, small (0.2), medium (0.5), and large (0.8) [43]. The Cochran's* Q* homogeneity statistic was used to determine the heterogeneity of the mean ESs across the groups. Moderator analyses were conducted to test the ES difference among the categorical subgroups of each moderator. The mean ES and 95 CI of each subgroup was also examined to see if an exercise intervention has an effect greater than zero. In addition, we examined the funnel plots, the Duval and Tweedie's trim and fill method, and Egger's test to detect the publication bias as all studies were published in the peer-reviewed journals in which the results are possibly subjected to publication bias.

## 3. Results

### 3.1. Search Results

Figure 1 outlines the flow diagram of the study selection process. The literature search identified 91 articles. We next reviewed the articles in full text to determine final eligibility. 17 studies ultimately met the eligibility criteria providing sufficient information for computing ESs. Some of the studies included results from separate, independent trials testing the effects of two or more exercise modalities on EF, so the 17 studies yielded a total of 34 ESs for the final meta-analysis.

### 3.2. Publication Bias

The funnel plot examination showed that the publication bias had little influence on our result. The studies included in the present meta-analysis were symmetrically distributed around the mean ES. The Duval and Tweedie's trim and fill method also predicted no missing study to this meta-analysis. However, the regression intercept (3.37) from Egger's test results was statistically significant (*p* = 0.001) that a potential publication bias for this meta-analysis should be noted for its interpretation.

### 3.3. Study Characteristics

The characteristics of these studies are shown in Table 1. Studies from around the world were included, including North America, Australia, South America, Europe, and Asia. They included women and men (mean age = 47.23) who are overweight or obesity with or without comorbidity. One trial used only men, 17 trials used only females, and 16 trials used both sexes. The included studies that accounted for other potential confounders such as smoking. 16 of 34 trials in 17 studies were RCTs with no exercise intervention control group.  Table 2 shows the characteristics of interventions within the included studies. 71% of trials (*n* = 24 trials) incorporated aerobic exercise; 18% (*n* = 6 trials) and 11% (*n* = 4 trials) of trials incorporated resistance exercise and combined with aerobic and resistance exercise, respectively.  Table 3 shows FMD protocol and outcomes. 23 trials in 17 studies reported fasting time (ranged from 0.5 hours to overnight fasting). Brachial artery FMD was measured in all of the included studies. 71% studies reported mean and SD of FMD percentage at preintervention and postintervention, and the rest of the studies reported amount of change or 95% confidence interval (CI). The mean and SD of FMD was 7.15% ± 3.05 (range 2.7 to 11.28%) before intervention and 8.67% ± 2.62 (range 4 to 12.9%) after intervention, and rate of change for FMD was 1.17% ± 1.63 (range −1.3 to 5%) in the treatment group. In the control group, the mean and SD of FMD was 5.69% ± 2.12 (range 2.5 to 9.9%) before intervention and 6.65% ± 2.46 (range 3.8 to 10.1%) after intervention, and rate of change for FMD was 0.61% ± 2.16 (range −0.7 to 4.9%).

### 3.4. Quality of Included Studies

Quality score by the PEDro scale was 7.2 ± 1.5 (ranged from 5 to 9), the median score of 8 of a maximum possible of 10 (Table 4). This score is equivalent to moderate to high quality [42]. All of the studies satisfied the following criteria: baseline comparability, intention-to-treat analysis, and mean and variability data. No study reported having blinded therapists.

### 3.5. Overall Effect Size

**Figure 2:**
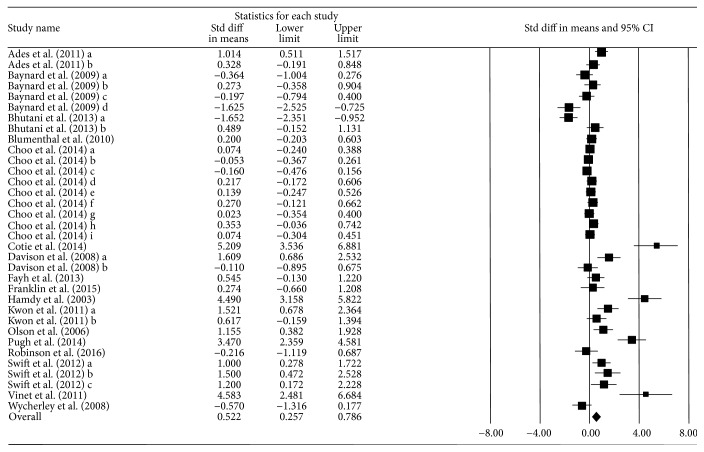
Forest plot illustrating effect of exercise intervention on FMD.

The overall mean ES was 0.522 (95% CI = 0.257, 0.786) and statistically significant. This quantitative synthesis yielded a medium and positive ES using a random effect model. This indicates that exercise training is effective in improving FMD in overweight and obese adults. There was observed to be heterogeneous (*Q*_*b*_ = 239, *p* ≤ 0.001, *I*^2^ = 86.19), suggesting that moderator analyses are needed to better understand the exercise intervention effect on FMD.  Figure 2 provides Forest plot with ESs of the analysis results.

### 3.6. Moderator Analysis

The moderator analyses were performed to examine the effect of body weight change, diet intervention, exercise modality, comorbidity, and baseline BMI. The results demonstrated that only comorbidity status explained the heterogeneity of ESs (*Q*_*b*_ = 6.39, df = 1, and *p* = 0.011).  Table 5 shows the results of moderator analyses, which includes ESs, CIs, and Cochran's *Q* statistics for each moderator variables. A large ES was found in the combination exercise, low intensity exercise, and comorbidity subgroups (ES = 0.82~1.24). A moderate to large ES was found in body weight loss, with and without diet intervention, more than 12-week exercise duration, aerobic exercise, and between 30 and 34.9 baseline BMI (ES = 0.51~0.71).

## 4. Discussion

In this meta-analysis, we found 34 trials from 17 studies including 1,045 overweight and obese adults. The meta-analysis result showed that exercise training significantly improves vascular function as measured by FMD of the brachial artery. The studies were randomized controlled trials of control and noncontrol groups of Asian and Western adult populations. Endothelial dysfunction is inherent in overweight and obese adults, and exercise training is universally accepted to ameliorate the obesity-associated endothelial dysfunction in healthy adults [16, 44]; however, more examination of the specific effects of exercise training on EF in overweight and obese population is still needed. Therefore, we combined data from each of the clinical trials to understand the relationship between exercise training and EF in overweight and obese adults.

Our results demonstrated that exercise has a moderate benefit on the improvement of FMD on overweight and obese adult populations in exercise intervention studies. When we probed moderators to examine the possible associations with ESs, we found that only comorbidity status influences the effectiveness of exercise intervention on EF. To our knowledge, we are the first to report this result. The finding could explain why exercise may not reverse the reduction of FMD attributable solely to obesity in isolation, whereas exercise may reverse the portion attributable to a comorbidity. While the explanation contrasts two previous meta-analyses [16, 45] showing that exercise is an effective method to improve FMD, they use pooled data from both obese and nonobese groups. If obesity acts on FMD in an exercise-independent way, it is possible that our finding of an FMD improvement results from the influence of exercise intervention on the portion of FMD decrement attributable to the comorbidity, rather than on the portion of FMD decrement attributable to obesity. We were unable to extrapolate the underlying mechanism of this finding, because it is beyond the scope of our study. Further research is required to examine different populations according to study characteristics (age, type of disease, stage of disease, exercise intensity, exercise type, treatment modality, etc.).

The examination of mean ES and 95 CI of each subgroup showed that ES is above medium in the subgroups with weight loss whereas there is no significant benefit of exercise intervention in the weight gain group. Although the mechanism of the effects of weight loss on FMD in overweight and obese adults requires more elucidation, numerous studies confirm the positive effects of weight loss by exercise on FMD. The beneficial effect of weight reduction by lifestyle changes, such as exercise to improve vascular function in obese adults, is strongly supported in [46], and a meta-analysis of the relationship between weight change and EF has reported a positive correlation between weight loss and an increase in FMD [45]. Therefore, it is speculated that weight reduction may be a major factor enhancing FMD in obese individuals and may depend on the method of weight reduction.

A study of the effect of surgically induced weight loss on FMD in hypertensive obese patients showed that bariatric surgery-induced weight loss improves blood pressure (BP), high-sensitivity C-reactive protein (hs-CRP), leptin, homeostasis model assessment (HOMA-IR), and abdominal fat, whereas FMD does not improve [47]. A study examining the effects of dietary weight loss on vascular function in obese men demonstrated that diet-induced weight reduction decreases aortic stiffness, total and low-density lipoprotein cholesterol, triglycerides, insulin resistance, and BP without alteration of FMD [48]. Similarly, our results demonstrate the significant benefit of exercise on FMD in overweight and obese adults regardless of diet control. Taken together, the above results indicating that exercise-mediated weight loss may improve FMD, but not diet control or surgery, suggest that exercise is a key regulator of FMD in overweight and obese adults. The notion that exercise-induced shear stress improves FMD in overweight and obese adults through an increase in the activity and expression of endothelial nitric oxide synthase (eNOS) that augments NO bioavailability also supports the suggestion [49–51].

We also found a moderate to large beneficial effect of exercise in the longer-period intervention subgroup than with 12 weeks of exercise program, and no significant benefit in the group with less than 12 weeks of intervention. We suggest that at least 12 weeks of exercise intervention may improve FMD in overweight and obese adults. Although previous reviews hypothesized that a longer duration may increase efficacy and maintain the effect on EF from the exercise intervention [16, 44], our meta-analysis provides the first quantitative evidence of the optimal exercise intervention duration to improve EF in overweight and obese adults.

As mentioned, exercise modality and intensity to improve EF remains controversial. For example, a meta-analysis of obese and nonobese adults showed that any type of exercise, including resistance, aerobic, and combined training improves EF [16]; however, another meta-analysis demonstrated that resistance exercise associates with increased arterial stiffness [18]. Our results confirmed no effect in the resistance exercise, medium to large effect in the aerobic exercise, and large effect in the combined exercise. We interpret our findings with caution, because more than 70% of included studies utilized aerobic exercises as an intervention modality. Furthermore, a recent study showed that high intensity exercise improves FMD more than moderate intensity, because higher intensity exercise causes greater shear stress resulting in more NO activation [17], even though other studies reported that high intensity exercise significantly reduces FMD [19, 20]. Our meta-analysis results showed that high intensity exercise has no effect, whereas low and moderate intensity exercise have large and medium ESs, respectively. Again, we interpret the results with caution, because a limited number of studies reported results for high (*n* = 1) and low (*n* = 3).

Moderator analysis also demonstrated that adults with a BMI 30–34.9 (level 1 obesity) have large and medium to large beneficial effect from exercise, respectively, whereas adults without a comorbidity and BMI < 30 or ≥35 have no significant benefit from exercise. Previously, Joris et al. [45] reported that the effects on FMD linearly relate to amount of weight loss in groups with obesity-related morbidities compared with healthy adults. Since this study pooled obese and nonobese adults and it did not break down groups by method of weight loss, a direct comparison with our study is not possible, because our data include only obese adults [45].

The effect of exercise on FMD may also depend on baseline BMI. Our result showed that only adults with level 1 obesity have a benefit from exercise training on FMD. A meta-analysis by Ashor et al., however, showed a greater effect of exercise on FMD in nonobese individuals than in obese individuals [16]. Again, a direct comparison is not possible. Further research should clarify the relationship between baseline obesity and exercise effect on FMD.

This study must be interpreted in the context of multiple limitations. First, the range in FMD levels in the studies is relatively small, and there is substantially less data for those with BMI > 35. Second, there were methodological limitations. FMD is well known for being operator and protocol dependent, and there was considerable variation in FMD data collection methodology [52, 53]. Therefore, further well-controlled studies are needed to draw accurate conclusions.

In summary, our meta-analysis indicates that exercise training is able to improve EF in overweight and obese adults, and that the effect of exercise may depend on the different characteristics of exercise intervention and on participants' demographics.

## Figures and Tables

**Table 1:** Baseline characteristics of the included studies.

Author and year	County	Subject characteristics of treatment group						Subject characteristics of control group						Smoker
		Sample size	Gender	Age	Health status	BMI	Δ weight	Sample size	Gender	Age	Health status	BMI	Δ weight	
Ades et al. (2011) a [23]	USA	23	Both	66	Coronary heart disease	32	−8							Not included
Ades et al. (2011) b [23]	USA	15	Both	62	Coronary heart disease	33	−2							Not included
Baynard et al. (2009) a [24]	USA	10	Both	52	Metabolic syndrome	34	0							Not included
Baynard et al. (2009) b [24]	USA	10	Both	52	Metabolic syndrome	34	0							Not included
Baynard et al. (2009) c [24]	USA	11	Both	53	Healthy	33	0							Not included
Baynard et al. (2009) d [24]	USA	11	Both	53	Healthy	33	0							Not included
Bhutani et al. (2013) a [25]	USA	18	Both	45	Healthy	35	−6	25	Both	42	Healthy	35	−3	Not included
Bhutani et al. (2013) b [25]	USA	24	Both	42	Healthy	35	−1	16	Both	49	Healthy	35	0	Not included
Blumenthal et al. (2010) [26]	USA	49	Both	52	HTN	34	−9	46	Both	52	HTN	33	0	Included
Choo et al. (2014) a [27]	South of Korea	39	Female	42	Healthy	29	−2							Included
Choo et al. (2014) b [27]	South of Korea	39	Female	42	Healthy	29	−2							Included
Choo et al. (2014) c [27]	South of Korea	39	Female	42	Healthy	29	−2							Included
Choo et al. (2014) d [27]	South of Korea	26	Female	46	Healthy	28	−2							Included
Choo et al. (2014) e [27]	South of Korea	26	Female	46	Healthy	28	−2							Included
Choo et al. (2014) f [27]	South of Korea	26	Female	46	Healthy	28	−1							Included
Choo et al. (2014) g [27]	South of Korea	27	Female	42	Healthy	29	−1							Included
Choo et al. (2014) h [27]	South of Korea	27	Female	42	Healthy	29	−1							Included
Choo et al. (2014) i [27]	South of Korea	27	Female	42	Healthy	29	−1							Included
Cotie et al. (2014) [28]	Canada	20	Female	30	Healthy	32	−6							NR
Davison et al. (2008) a [29]	Australia	13	Both	45	Healthy	34	1	11	Both	44	Healthy	35	2	
Davison et al. (2008) b [29]	Australia	13	Both	46	Healthy	33	1	12	Both	45	Healthy	33	−2	
Fayh et al. (2013) [30]	Brazil	17	Both	31	Healthy	35	−4	18	Both	32	Healthy	35	−5	Not included
Franklin et al. (2015) [31]	USA	10	Female	30	Healthy	34	−1	8	Female	31	Healthy	32	0	Not included
Hamdy et al. (2003) [32]	USA	24	Both	49	Insulin resistance syndrome	37	−7							Not included
Kwon et al. (2011) a [33]	South of Korea	13	Female	56	Type 2 diabetes	27	−2	15	Female	59	Type 2 Diabetes	27	−1	NR
Kwon et al. (2011) b [33]	South of Korea	12	Female	56	Type 2 diabetes	27	−1	15	Female	59	Type 2 Diabetes	27	−1	NR
Olson et al. (2006) [34]	USA	15	Female	38	Healthy	28	2	15	Female	38	Healthy	28	0	Not included
Pugh et al. (2014) [35]	UK	13	Both	50	Nonalcoholic fatty liver disease	30	−2	8	Both	47	Healthy	30	−1	
Robinson et al. (2016) [36]	USA	10	Both	34	Healthy	32	−3	9	Both	28		33	0	Not included
Swift et al. (2012) a [37]	USA	68	Female	57	Elevated BP	32	−1	23	Female	57	Elevated BP	32	−1	
Swift et al. (2012) b [37]	USA	32	Female	56	Elevated BP	33	−1	23	Female	57	Elevated BP	32	−1	
Swift et al. (2012) c [37]	USA	32	Female	56	Elevated BP	31	−1	23	Female	57	Elevated BP	32	−1	
Vinet et al. (2011) [38]	France	10	Male	51	Healthy	33	−2							Not included
Wycherley et al. (2008) [39]	Australia	13	Both	52	Type 2 diabetes	34	−8	16	Both	53	Type 2 Diabetes	35	−9	

*Note*. NR: no report.

**Table 2:** Characteristics of intervention of the included studies.

Author and year	Exercise intervention					Additional diet intervention
	Type	Duration (weeks)	Frequency of sessions (per week)	Duration of session (min)	Intensity	
Ades et al. (2011) a [23]	Aerobic	16	1–3	40–60	Low (high-caloric-expenditure)	Yes
Ades et al. (2011) b [23]	Aerobic	16	1–3	25–40	Higher (lower-caloric-expenditure)	Yes
Baynard et al. (2009) a [24]	Aerobic	10 days	6	60	70–75% of VO_2_ peak	No
Baynard et al. (2009) b [24]	Aerobic	10 days	6	60	70–75% of VO_2_ peak	No
Baynard et al. (2009) c [24]	Aerobic	10 days	6	60	70–75% of VO_2_ peak	No
Baynard et al. (2009) d [24]	Aerobic	10 days	6	60	70–75% of VO_2_ peak	No
Bhutani et al. (2013) a [25]	Aerobic	12	3	24–40	60–75% of HRmax	Yes
Bhutani et al. (2013) b [25]	Aerobic	12	3	24–40	60–75% of HRmax	No
Blumenthal et al. (2010) [26]	Aerobic	16	3	45	70–85% of HRR	Yes
Choo et al. (2014) a [27]	Aerobic	12	3	60	50–70% of HRR	Yes
Choo et al. (2014) b [27]	Aerobic	24	3	60	50–70% of HRR	Yes
Choo et al. (2014) c [27]	Aerobic	38	3	60	50–70% of HRR	Yes
Choo et al. (2014) d [27]	Resistance	12	3	60	40–60% of MS	Yes
Choo et al. (2014) e [27]	Resistance	24	3	60	40–60% of MS	Yes
Choo et al. (2014) f [27]	Resistance	38	3	60	40–60% of MS	Yes
Choo et al. (2014) g [27]	Combined	12	3	60 (30 + 30)	50–70% of HRR 40–60% of MS	Yes
Choo et al. (2014) h [27]	Combined	24	3	60 (30 + 30)	50–70% of HRR 40–60% of MS	Yes
Choo et al. (2014) i [27]	Combined	38	3	60 (30 + 30)	50–70% of HRR 40–60% of MS	Yes
Cotie et al. (2014) [28]	Combined	16	7	Expend 250 kal/day	70%/3 set 10 rep	Yes
Davison et al. (2008) a [29]	Aerobic	12	At least 1	45	75% of HRmax	Yes
Davison et al. (2008) b [29]	Aerobic	12	At least 1	45	75% of HRmax	Yes
Fayh et al. (2013) [30]	Aerobic	10	3	45	70% of HRmax	Yes
Franklin et al. (2015) [31]	Circuit-based RT	8	2		80–90% of 10 RM	No
Hamdy et al. (2003) [32]	Aerobic	24	3	30	60–80% of HRmax	Yes
Kwon et al. (2011) a [33]	Aerobic	12	5	60	Moderate (3.6–6 METs)	No
Kwon et al. (2011) b [33]	Resistance	12	5	60	Bands provide 1.2–3.2 kg of resistance	No
Olson et al. (2006) [34]	Resistance	1 year	At least 2		3 sets 8–10 repetitions	No
Pugh et al. (2014) [35]	Aerobic	12	5	45	60% of HRR	Yes
Robinson et al. (2016) [36]	Aerobic	8	3	30–45	75% of HRmax	No
Swift et al. (2012) a [37]	Aerobic	24	3-4	Expend 4 kcal/kg	50% of VO_2_ peak	No
Swift et al. (2012) b [37]	Aerobic	24	3-4	Expend 8 kcal/kg	50% of VO_2_ peak	No
Swift et al. (2012) c [37]	Aerobic	24	3-4	Expend 12 kcal/kg	50% of VO_2_ peak	No
Vinet et al. (2011) [38]	Aerobic	8	3	45	LIPOXmaxHR ± 5	No
Wycherley et al. (2008) [39]	Aerobic	12	4-5	50–60	60–80% of HRmax	Yes

*Note*. HRR: Heart Rate Reserve, MS: Maximum Strength, LIPOXmaxHR: maximum lipid-oxidation point.

**Table 3:** FMD protocol and outcomes.

Author anB3:H25	Timing of measurement	Placed cuff	Treatment group				Control group			
			Preintervention		Postintervention		Preintervention		Postintervention	
			mean	SD	mean	SD	mean	SD	mean	SD
Ades et al. (2011) a [23]	Fast	Brachial artery	2.9	3.6	6.5	3.5				
Ades et al. (2011) b [23]	Fast	Brachial artery	3.6	4.1	4.9	3.8				
Baynard et al. (2009) a [24]	Overnight fast	Brachial artery	8	1.5	7.5	1.2				
Baynard et al. (2009) b [24]	0.5–1 h ingestion	Brachial artery	6	1.1	6.3	1.1				
Baynard et al. (2009) c [24]	Overnight fast	Brachial artery	10.4	1.1	10.2	0.9				
Baynard et al. (2009) d [24]	0.5–1 h ingestion	Brachial artery	9.8	0.8	8.5	0.8				
Bhutani et al. (2013) a [25]	NR	Brachial artery	3.8	1.2	6.4	0.8	4.8	1.2	9.7	1.8
Bhutani et al. (2013) b [25]	NR	Brachial artery	6.8	1.3	7.2	1.4	7	3.3	6.3	3
Blumenthal et al. (2010) [26]	Overnight fast	Brachial artery			4^z^	1			3.8^z^	1
Choo et al. (2014) a [27]	Overnight fast	Brachial artery	11.28	3.5	11.55	3.8				
Choo et al. (2014) b [27]	Overnight fast	Brachial artery	11.28	3.5	11.08	4.05				
Choo et al. (2014) c [27]	Overnight fast	Brachial artery	11.28	3.5	10.7	3.75				
Choo et al. (2014) d [27]	Overnight fast	Brachial artery	10.32	3.8	11.22	4.43				
Choo et al. (2014) e [27]	Overnight fast	Brachial artery	10.32	3.8	10.89	4.33				
Choo et al. (2014) f [27]	Overnight fast	Brachial artery	10.32	3.8	11.54	4.99				
Choo et al. (2014) g [27]	Overnight fast	Brachial artery	11.02	3.49	11.1	3.4				
Choo et al. (2014) h [27]	Overnight fast	Brachial artery	11.02	3.49	12.41	4.27				
Choo et al. (2014) i [27]	Overnight fast	Brachial artery	11.02	3.49	11.3	4.04				
Cotie et al. (2014) [28]	NR	Brachial artery	4	0.5	6.9	0.6				
Davison et al. (2008) a [29]	NR	Brachial artery	5.37	0.68	−0.4^a^	0.77^b^	3.65	1.4	−0.3^a^	0.53^b^
Davison et al. (2008) b [29]	NR	Brachial artery	4.05	0.51	1.5^a^	0.68^b^	4.12	0.75	1.8^a^	0.89^b^
Fayh et al. (2013) [30]	Overnight fast	Brachial artery	8.1	3.6	10.7	3.6	9.9	3.4	10.1	5.8
Franklin et al. (2015) [31]	NR	Brachial artery	9.5	1.6	9.8	1.6	8.4	3.5	8	3.3
Hamdy et al. (2003) [32]	NR	Brachial artery	7.9	1	12.9	1.2				
Kwon et al. (2011) a [33]	10 h fast	Brachial artery	4.3	1.6	6.4	1.9	4.7	1.9	4	1.9
Kwon et al. (2011) b [33]	10 h fast	Brachial artery	4.9	2.5	5.6	2.8	4.7	1.9	4	1.9
Olson et al. (2006) [34]	Overnight fast	Brachial artery	6.3	0.2	6.2	0.1	6.3	0.2	6	0.1
Pugh et al. (2014) [35]	NR	Brachial artery	4.79		8.57	(2.24–4.71)^c^	5.94		5.32	(−1.72–1.46)^c^
					3.47^a^				−0.13^a^	
Robinson et al. (2016) [36]	NR	Brachial artery	8.6	4.8	7.7	2.79	9.3	4.2	9.3	4.1
Swift et al. (2012) a [37]	Fast	Brachial artery	4	2.6	1^a^	(0.29–1.76)^c^	4.7	2.4	−0.5^a^	(−1.79–0.74)^c^
Swift et al. (2012) b [37]	Fast	Brachial artery	4.4	2.4	1.5^a^	(0.48–2.62)^c^	4.7	2.4	−0.5^a^	(−1.79–0.74)^c^
Swift et al. (2012) c [37]	Fast	Brachial artery	3.7	2.6	1.2^a^	(0.1–2.24)^c^	4.7	2.4	−0.5^a^	(−1.79–0.74)^c^
Vinet et al. (2011) [38]	Overnight fast	Brachial artery	2.7	0.4	4.8	0.5				
Wycherley et al. (2008) [39]	Fast	Brachial artery	4.2	1.2	−0.52^a^	1.06^b^	2.5	0.9	0.03^a^	0.26^b^

*Note*. ^a^Δmean; ^b^ΔSD; ^c^95% confidence interval; ^z^adjusted for value of preintervention; NR: no report.

**Table 4:** Methodological scores by Physiotherapy Evidence Database (PEDro) scale.

Studies	PEDro criterion											Total score
	1	2	3	4	5	6	7	8	9	10	11	
Ades et al. (2011) [23]	1	1	1	1	1	0	0	1	1	1	1	9
Baynard et al. (2009) [24]	1	0	0	1	0	0	0	1	1	1	1	6
Bhutani et al. (2013) [25]	1	1	1	1	0	0	0	0	1	1	1	7
Blumenthal et al. (2010) [26]	1	1	1	1	0	0	0	1	1	1	1	8
Choo et al. (2014) [27]	1	1	1	1	1	0	1	0	1	1	1	9
Cotie et al. (2014) [28]	0	0	0	1	0	0	0	1	1	1	1	5
Davison et al. (2008) [29]	1	1	1	1	1	0	1	0	1	1	1	9
Fayh et al. (2013) [30]	1	1	1	1	0	0	0	0	1	1	1	7
Franklin et al. (2015) [31]	1	1	1	1	0	0	0	1	1	1	1	8
Hamdy et al. (2003) [32]	1	0	0	1	0	0	0	0	1	1	1	5
Kwon et al. (2011) [33]	1	1	1	1	0	0	0	1	1	1	1	8
Olson et al. (2006) [34]	1	1	1	1	0	0	1	1	1	1	1	9
Pugh et al. (2014) [35]	1	1	1	1	0	0	0	0	1	1	1	7
Robinson et al. (2016) [36]	1	0	0	1	0	0	0	0	1	1	1	5
Swift et al. (2012) [37]	1	1	1	1	0	0	1	0	1	1	1	8
Vinet et al. (2011) [38]	1	0	0	1	0	0	0	1	1	0	1	5
Wycherley et al. (2008) [39]	1	1	1	1	0	0	0	1	1	1	1	8

*Total*	16	12	12	17	3	0	4	9	17	16	17	

*Note*. Each number of PEDro criterion is represented as follows: 1: inclusion and source; 2: random allocation; 3: concealed allocation; 4: baseline comparability; 5: blinded subjects; 6: blinded therapists; 7: blinded assessors; 8: outcomes for >85%; 9: intention-to-treat analysis; 10: between-group comparisons; 11: mean and variability data.

**Table 5:** Subgroup analysis.

Moderator variable	*n*	ES	95% CI		*Q*_*b*_
			Lower	Upper	
Body weight change					
Increase	7	0.10	−0.50	0.70	2.545
0–2.9 kg loss	19	0.61	0.26	0.97	
≥3 kg loss	8	0.70	0.12	1.28	
Diet intervention					
Yes	20	0.51	0.18	0.85	0.008
No	14	0.54	0.11	0.97	
Exercise duration					
<12 weeks	8	0.09	−0.50	0.68	2.868
12–23 weeks	15	0.61	0.20	1.02	
≥24 weeks	11	0.71	0.24	1.18	
Exercise type					
Resistance	6	0.43	−0.22	1.07	0.601
Aerobic	24	0.52	0.18	0.85	
Combined	4	0.82	0.01	1.62	
Exercise intensity					
Low	3	1.24	0.29	2.19	2.401
Moderate	30	0.47	0.19	0.75	
High	1	0.27	−1.38	1.93	
Comorbidity					
No	21	0.26	−0.06	0.58	6.392^*∗*^
Yes	13	0.95	0.52	1.37	
Baseline BMI					
25–29.9	12	0.31	−0.11	0.73	1.647
30–34.9	19	0.67	0.30	1.04	
≥35	3	0.66	−0.28	1.61	

*Note*. ^*∗*^*p* < 0.05.
